# Heart Failure Telemonitoring in Japan and Sweden: A Cross-Sectional Survey

**DOI:** 10.2196/jmir.4825

**Published:** 2015-11-13

**Authors:** Naoko P Kato, Peter Johansson, Ikuko Okada, Arjen E de Vries, Koichiro Kinugawa, Anna Strömberg, Tiny Jaarsma

**Affiliations:** ^1^ Department of Social and Welfare Studies Faculty of Health Sciences Linköping University Norrköping Sweden; ^2^ Department of Therapeutic Strategy for Heart Failure The University of Tokyo Graduate School of Medicine Tokyo Japan; ^3^ JSPS Postdoctoral Fellow for Research Abroad Tokyo Japan; ^4^ Department of Cardiology County Council of Östergötland Linköping Sweden; ^5^ Department of Cardiology University Medical Center Groningen Groningen Netherlands; ^6^ Department of Medical and Health Sciences, Division of Nursing Science, and Department of Cardiology Linköping University Linköping Sweden

**Keywords:** disease management, expectation, heart failure, implementation barriers, nurses, perception, physicians, telemedicine

## Abstract

**Background:**

Telemonitoring of heart failure (HF) patients is increasingly discussed at conferences and addressed in research. However, little is known about actual use in specific countries.

**Objective:**

We aimed to (1) describe the use of non-invasive HF telemonitoring, (2) clarify expectations of telemonitoring among cardiologists and nurses, and (3) describe barriers to the implementation of telemonitoring in Japan and Sweden.

**Methods:**

This study used a cross-sectional survey of non-invasive HF telemonitoring. A total of 378 Japanese (120 cardiologists, 258 nurses) and 120 Swedish (39 cardiologists, 81 nurses) health care professionals from 165 Japanese and 61 Swedish hospitals/clinics nationwide participated in the study (210 in Japan and 98 in Sweden were approached). Data were collected between November 2013 and May 2014 with a questionnaire that was adapted from a previous Dutch study on telemonitoring.

**Results:**

The mean age of the cardiologists and nurses was 47 years and 41 years, respectively. Experience at the current position caring for HF patients was 19 years among the physicians and 15 years among the nurses. In total, 7 Japanese (4.2%) and none of the Swedish health care institutions used telemonitoring. One fourth (24.0%, 118/498) of the health care professionals were familiar with the technology (in Japan: 21.6%, 82/378; in Sweden: 30.0%, 36/120). The highest expectations of telemonitoring (rated on a scale from 0-10) were reduced hospitalizations (8.3 in Japan and 7.5 in Sweden), increased patient self-care (7.8 and 7.4), and offering high-quality care (7.8 and 7.0). The major goal for introducing telemonitoring was to monitor physical condition and recognize signs of worsening HF in Japan (94.1%, 352/374) and Sweden (88.7%, 102/115). The following reasons were also high in Sweden: to monitor effects of treatment and adjust it remotely (86.9%, 100/115) and to do remote drug titration (79.1%, 91/115). Just under a quarter of Japanese (22.4%, 85/378) and over a third of Swedish (38.1%, 45/118) health care professionals thought that telemonitoring was a good way to follow up stable HF patients. Three domains of barriers were identified by content analysis: organizational barriers “how are we going to do it?” (categories include structure and resource), health care professionals themselves “what do we need to know and do” (reservation), and barriers related to patients “not everybody would benefit” (internal and external shortcomings).

**Conclusions:**

Telemonitoring for HF patients has not been implemented in Japan or Sweden. However, health care professionals have expectations of telemonitoring to reduce patients’ hospitalizations and increase patient self-care. There are still a wide range of barriers to the implementation of HF telemonitoring.

## Introduction

Management of heart failure (HF) poses substantial challenges to health care systems worldwide. Advances in modern telecommunication technologies have created new opportunities to provide telemedical care as an adjunct to the management of HF patients. Telemonitoring defined as “the remote monitoring of patients including the use of audio, video, other telecommunications, and electronic information processing technologies to monitor patient status at a distance” [1] might be an option for future HF management.

In several meta-analyses, telemonitoring for HF patients has been shown to reduce mortality and hospital admissions [2-4]. However, findings from recent studies are not consistent [5-8], raising doubts about the potential of telemonitoring in HF management. Because of this conflicting evidence, the HF guideline committee of the European Society of Cardiology has not recommended use of telemonitoring in regular HF care [9]. HF telemonitoring is, however, considered to be promising, and many HF telemonitoring studies are underway. In contrast, little is known about HF telemonitoring in daily practice. Only a few studies have examined the actual usage rate of telemonitoring, health care professionals’ expectations of telemonitoring, and practical issues related to telemonitoring [10-12]. These aspects are vital for HF telemonitoring in future practice.

Sweden and Japan are two countries with advanced information technology, where HF management is a medical and financial challenge [13,14]. Findings on implementation and attitudes to telemonitoring in these two countries might make it possible to predict how HF telemonitoring will be used in other European and Asian countries in the near future. In addition, clarifying practical issues related to HF telemonitoring could lead to solutions for using the device in daily practice.

This study therefore aimed to (1) describe the use of HF telemonitoring, (2) clarify expectations of telemonitoring among cardiologists and nurses, and (3) describe barriers to the implementation of telemonitoring in Japan and Sweden. The findings were compared between the two countries, which could facilitate generalizability.

## Methods

### Design and Definition

This study used a cross-sectional survey of non-invasive HF telemonitoring. Non-invasive HF telemonitoring was defined as the remote, Internet-based monitoring of HF patients on weight, blood pressure, heart rate, and signs and symptoms that disclose the actual condition of HF patient [11]. The devices are used by the patient in their own home environment, and the generated data are communicated by the Internet to a telemonitoring center. Health care professionals receive the data in the center and provide feedback to the patients. Telemonitoring by means of telephone, telephone support, telephone follow-up, or implantable devices was not included in this study as our focus was to investigate perceptions of using telemonitoring devices that required active user interaction.

### Study Procedure and Participants

Physicians and nurses working with HF patients in Japan and Sweden participated in this study. First, we made a list of potential hospitals. In Japan, a total of 210 public and private hospitals nationwide were randomly extracted from 994 hospitals that were recognized training facilities for cardiologists by the Japanese Circulation Society, based on each prefecture population. At least two hospitals from each of 47 prefectures (one university and one non-university hospital) were included. In Sweden, all 98 hospitals/primary care centers with a specialist HF clinic in all 21 county councils and regions were included.

Between November 2013 and May 2014, the questionnaire was sent to hospital departments responsible for HF patient care, for example, cardiology departments, HF clinics, or primary care centers with a specialized HF nurse, along with an information letter requesting their help. For each hospital/clinic, it was requested that the questionnaire be completed by 1 physician and 2 nurses working with HF patients on a regular basis. The questionnaires were given a code for each hospital/clinic so that it was possible to tell which hospitals returned the questionnaire. Respondent confidentiality was assured by omitting names and other personal information in the questionnaire.

### Instrument

To clarify health care providers’ perceptions of HF telemonitoring, a questionnaire previously developed by de Vries et al was used [11]. Face validity of the original questionnaire has been confirmed by 10 cardiologists and 10 HF nurses. The original instrument was developed in Dutch [11] and then translated into English. In our study, 3 researchers who were native speakers of Dutch, Japanese, and Swedish respectively, translated the instrument from English into Japanese and from English to Swedish, while carefully verifying the semantic equivalence of the translation. Where there were difficulties understanding the intent of the original questionnaire, the translated versions were checked against the original Dutch questionnaire.

To examine availability of telemonitoring, study participants were asked if they used telemonitoring for HF patients at that time. Those who did were asked about the system they used. As for awareness of telemonitoring, the participants were asked if they were familiar with HF telemonitoring. They responded to this question with yes or no. For data about the participants’ expectations of telemonitoring, we asked if the following four items could be main goals for telemonitoring: (1) monitoring physical condition and noticing declines, (2) monitoring effects of the treatment and adjusting it remotely, (3) remote drug titration, and (4) patient education. Again, the participants responded to four items with yes or no. They were also asked about good ways to follow up on stable HF patients. They responded to the 8 ways of follow-up including telemonitoring, outpatient clinic, and implantable telemonitoring device with yes or no. As for reasons for introducing telemonitoring in HF patients, health care professionals were asked to rate the importance level for introducing telemonitoring (eg, reduced readmission, increased patient self-care, high quality of care, and improved adherence to HF guidelines) on a 10-point scale (0= “not important”, 10=“very important”). We asked about barriers for implementation of telemonitoring in HF patients to health care professionals who had not used HF telemonitoring in their hospitals or clinics with open-ended question such as “What are the important barriers to use of telemonitoring in your institutions?”. Multimedia Appendix 1 shows the questionnaire used in the study.

We also examined characteristics of study participants, such as gender, age, experience of current position as physicians or nurses caring for HF patients, health care institutions, and computer skills and knowledge of, for instance, Word, Excel, and the Internet.

### Data Analysis

Data from all participants were included for analysis regardless of actual usage of HF telemonitoring at the time of the study. Descriptive analysis was used to present data. For continuous variables with a normal distribution, the mean and standard deviations are reported and were analyzed by Student *t* test to compare data between Japan and Sweden. For continuous variables not normally distributed, the median and interquartile range (Q1-Q3) are reported and were analyzed with Mann-Whitney U test. Categorical variables are presented with numbers and percentages and were analyzed with chi-square test and in a few cases, the Fisher’s exact test. All statistical tests were two-tailed, and statistical significance was defined as *P*<.05. All analyses were performed with SAS version 9.3.

Barriers for implementation of telemonitoring in HF patients were extracted by analyzing the participants’ responses to the open-ended questions in the questionnaire. All responses were transcribed verbatim and analyzed using content analysis methodology [15]. First, descriptions were divided into domains by 3 researchers (NPK, PJ, TJ), which was the highest conceptual level identified in the study. Subsequently, the descriptions were categorized into subcategories in each domain. Then, the subcategories were merged into categories, based on topic similarities. Where there was no consensus, discussions took place between the researchers until consensus was achieved.

## Results

### Participants and Response Rate

A total of 165 Japanese hospitals and 61 Swedish hospitals/clinics participated in this study. The response rate at hospital/clinic level (meaning that at least one health care provider answered the questionnaire) was 79% in Japan and 62% in Sweden. In total, data from 339 nurses (258 Japanese, 81 Swedish, response rate=55%), and 159 physicians (120 Japanese, 39 Swedish, response rate=52%) were analyzed in the study.

Table 1 shows the characteristics of the study participants. The mean age of the physicians and nurses was 47 years and 41 years, respectively. Japanese nurses were significantly younger than Swedish nurses (39 years vs 51 years, *P*<.001). Experience at the current position caring for HF patients was 19 years among physicians and 15 years among nurses. Almost every respondent (over 96%) had experience with standard programs such as Word and Excel and were able to use email and Internet.

**Table 1:** Characteristics of physicians and nurses.

	All physicians (n=159)	Japanese physicians (n=120)	Swedish physicians (n=39)	All nurses (n=339)	Japanese nurses (n=258)	Swedish nurses (n=81)
Male, n (%)	137 (86)	115 (96)	22 (56)	17 (6)	11 (5)	6 (8)
Age, mean (SD)	47 (8)	46 (7)	49 (9)	41 (10)	39 (8)	51 (9)
Experience at current position in years, mean (SD)	19.0 (8.1)	19.9 (7.5)	16.5 (9.3)	15.0 (8.7)	15.1 (8.4)	14.9 (9.7)
Time working with HF patients, hours/week, median (Q1-Q3)	12 (6-20)	15 (8-24)	10 (5-15)	30 (10-40)	40 (16-40)	14 (8-24)
University hospital, n (%)	61 (38)	56 (47)	5 (13)	113 (34)	109 (42)	4 (5)

### Availability of Telemonitoring for Heart Failure Patients

#### Actual Usage Rate of Telemonitoring for Heart Failure Patients

In total, 7 of 165 hospitals (4%) in Japan and none of the hospitals in Sweden used telemonitoring for HF patients at the time of the study. Of the Japanese hospitals, 6 used the same telemonitoring device (Karada Karute, Tanita Health Link, Inc.) since they were involved in a clinical trial of this equipment [16], and one hospital used devices made by Cyber Cross Japan Co. [17]. The functionality of the two systems used in Japan are similar. Physiological data generated at home, such as blood pressure and body weight, are transferred to a central Web server via the Internet. Nurses working at the center monitor the acquired data on a secure website 7 days a week. If the data exceed the acceptable range, the nurses in the center contact the patients to check the patients’ physical condition with the use of standard operating procedure. The patients were also contacted by physicians in accordance with the standard operating procedures [17,18].

#### Awareness

In total, 24.0% (118/498) of health care professionals (21.6%, 82/378 in Japan and 30.0%, 36/120 in Sweden) were familiar with HF telemonitoring. There was a significant difference (*P*<.001) in familiarity between physicians (49.6%, 63/159) and nurses (16.2%, 55/339).

### Expectations of Heart Failure Telemonitoring

#### Main Goals of Telemonitoring

**Figure 1:**
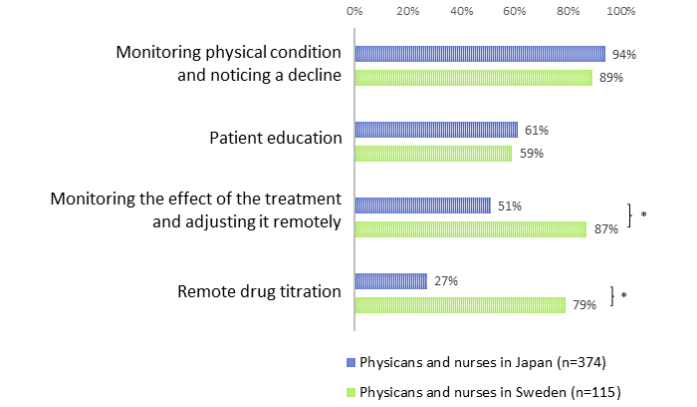
Main goals of heart failure telemonitoring according to the respondents. *P < .01 Japan vs Sweden by chi-square test.

As shown in Figure 1, the most frequent purpose for choosing HF telemonitoring in Japan (93.1%, 352/378) and Sweden (89.0%, 102/115) was “monitoring physical condition and noticing a decline”. In Japan, the second most frequent purpose was “patient education” (60.6%, 229/378). In Sweden, the second most frequent purpose was “monitoring effects of the treatment and adjusting it remotely” (86.9%, 100/115), whereas the third was “remote drug titration” (79.1%, 91/115). These two Swedish figures were significantly higher than the Japanese ones (both *P*<.001).

**Figure 2:**
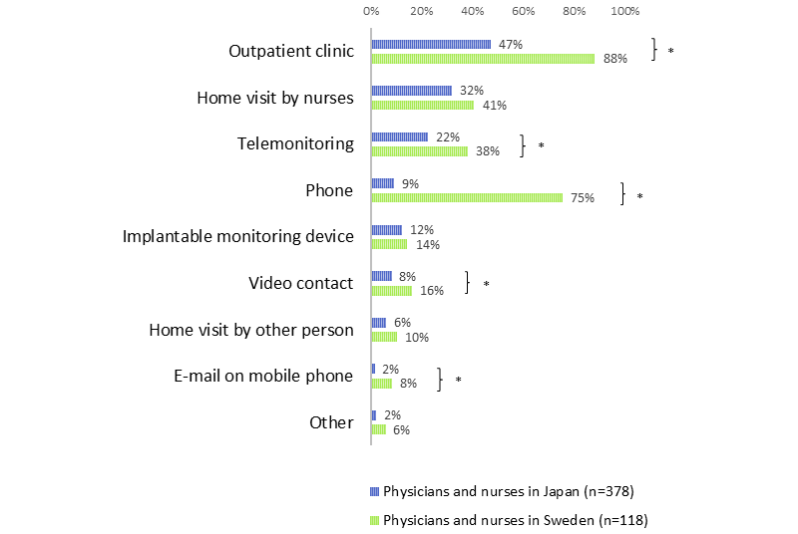
Good ways to follow up stable heart failure patients according to the respondents. *P < .01 Japan vs Sweden by chi-square test.

Figure 2 shows what health care providers think is a good way to follow up on stable HF patients. Just under a quarter of Japanese (22.4%, 85/378) and over a third of Swedish (38.1%, 45/118) health care providers thought that telemonitoring was a good way to follow up on stable HF patients. The percentage differed significantly between the two countries (*P*=.001). “Outpatient clinic” was the most frequently chosen option for a good way to follow up stable HF patients, regardless of country. In Japan, “Home visit by nurses” (31.7%, 120/378) was the second most common option, whereas in Sweden, “Phone” (75.4%, 89/118) was the second most common choice. “Implantable monitoring device” was chosen in 12.2% of Japanese (46/378) and 14.4% of Swedish (17/118) health care professionals. Approximately 10% of health care professionals selected other options including “Video contact”, “E-mail by mobile phone”, and “Home visit by other person”.

#### Reasons for Introducing Telemonitoring for Heart Failure Patients

**Figure 3:**
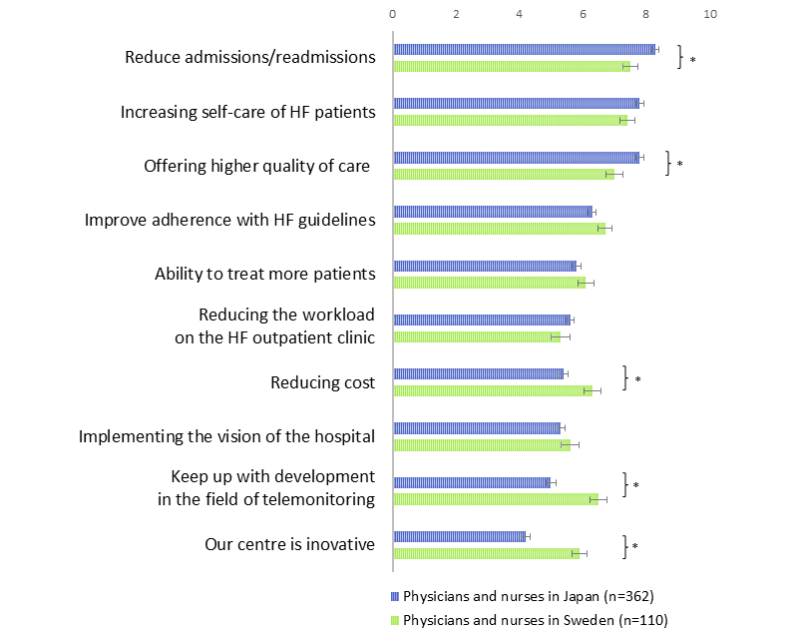
Expectations of heart failure telemonitoring. Importance level for introducing telemonitoring was evaluated on a 10-point scale (0= not important, 10= very important). Mean±SEM, *P <.05 Japan vs Sweden by Student *t* test.

Figure 3 represents the expectations of HF telemonitoring. Regardless of country, the top 3 reasons for introducing telemonitoring to HF patients were “Reduce patients’ admissions/readmissions” (Japan 8.3, SD 2.1; Sweden 7.5, SD 2.5), “Increasing patients’ self-care” (Japan 7.8, SD 2.1; Sweden 7.0, SD 2.5), and “Offering higher quality of care” (Japan 7.8, SD 2.3; Sweden 7.0, SD 2.7). The items “Ability to treat more patients”, “Reducing the work load on the HF clinic”, and “Reducing cost” were not rated as high (range from 5.3-6.3).

### Barriers to the Implementation of Telemonitoring for Heart Failure Patients

All answers from all 498 participants on the open question were considered in the analysis of the data on the question regarding barriers. These answers were condensed into categories and subcategories as presented in Tables 2-4. The quotes of some of the participants are used to illustrate the subcategory. Not many differences in the barriers to the implementation of HF telemonitoring were found between Sweden and Japan; therefore, they were summarized as one result. The barriers were divided into three domains: (1) organization, “How are we going to do it?”, (2) health care professionals, “What do we need to know and do?”, and (3) patients, “Not everybody would benefit”. The domain organization comprised two categories: resource and structures. In the health care professionals’ domain, the category reservation was extracted. In the patients’ domain, two categories (ie, internal and external shortcomings) were extracted.

**Table 2:** Barriers to implementation of HF telemonitoring based on the content analysis of the open-ended answers in the survey**—**Domain 1. Organization, “How are we going to do it?”.

Category	Subcategory	Quote
Resource	Manpower	We have a shortage of medical staff, we have no time (Sweden, physician, male)
		Difficulty securing medical staff who control overall data/system of telemonitoring (Japan, physician, male)
	Materials	There are no devices for HF telemonitoring (Japan, nurse, female)
		There are no adequate network systems in our hospital (Japan, physician, male)
	Funding and priority setting	We have no money for this (Sweden, physician, male)
		It depends on hospital policy (Japan, nurse, female)
Structure	Responsibilities	Who is responsible for telemonitoring? (Japan, physician, male)
		Which professionals play a key role? (Japan, nurse, female)
		What should we do if something happens? (Japan, nurse, male)
	Patients	What kind of patients can be a candidate for telemonitoring? (Japan, nurse, female)
		How many patients need telemonitoring? (Japan, nurse, female)
	Protocols	When or how often do we check data from patients? (Japan, nurse, female)
	Collaboration	Information-sharing among medical staff is not sufficient (Japan, nurse, female)
		It is difficult to collaborate with other hospitals/clinics (Sweden, physician, male)
	Safety	Support system and troubleshooting were not yet established (Japan, nurse, female)

**Table 3:** Barriers to implementation of HF telemonitoring based on the content analysis of the open-ended answers in the survey**—**Domain 2. Health care professionals, “What do we need to know and do?”.

Category	Subcategory	Quote
Reservation	Lack of advantage	I do not feel the need for telemonitoring (Sweden, nurse, female)
		We should prioritize self-care support rather than telemonitoring (Japan, physician, male)
	Skepticism about effects of telemonitoring/information technology	Some health care professionals have a resistance toward IT technology (Japan, nurse, female)
		There is no strong evidence of telemonitoring (Sweden, physician, female)
	Poor knowledge and skills of telemonitoring	I do not have enough confidence to explain telemonitoring to patients (Japan, nurse, female)
	Lack of computer skills	Some health care professionals do not have enough skills to use a personal computer (Japan, nurse, female)

**Table 4:** Barriers to implementation of HF telemonitoring based on the content analysis of the open ended answers in the survey**—**Domain 3. Patients, “Not everybody would benefit”.

Category	Subcategory	Quote
Internal shortcoming	Age-related conditions	Elderly patients cannot use the device (Japan, physician, male)
	Comorbidities	Patients with multiple diseases need additional monitoring (Sweden, nurse, female)
	Cognitive dysfunction	We cannot obtain correct information if patients have cognitive dysfunctions (Sweden, physician, female)
	Functional disability	Patients with impaired eyesight or hearing cannot use telemonitoring (Sweden, nurse, female)
		Patients with difficulty standing up cannot use telemonitoring (Japan, nurse, female)
	Anxiety/depression/worries	Patients may be greatly worried because of slightly changed data (Japan, nurse, female)
	No support	Patients cannot send physical data because of lack of family support (Japan, nurse, female)
	Lack of motivation	Patients who cannot measure their weight even now would not send their physical data by Internet (Japan, physician, male)
		Patients who do not have a cooperative relationship would not send data (Japan, nurse, female)
	Lack of acceptance	It is difficult to obtain acceptance of telemonitoring from patients (Japan, physician, female)
	Language problems	Non-Swedish speaking patients cannot understand the device (Sweden, nurse, female)
Environmental shortcoming	Lack of infrastructure	No/insufficient Internet connection and computers (Sweden, physician, female)
		Patients who are not used to using computers may not be able to use the device (Japan, nurse, female)
	Financial problems	Patients on welfare might not be able to pay the telemonitoring fee (Japan, nurse, female)

## Discussion

### Principal Findings

Despite increasing attention to telemonitoring for HF patients at professional conferences and some evidence being available in the literature [2-8], we found that only a few Japanese hospitals and no Swedish hospitals/clinics have introduced telemonitoring for HF patients. We also found that only a limited number (24%) of health care professionals were familiar with HF telemonitoring. However, at the same time we found that health care professionals could see a potential for using telemonitoring in their HF patients. In particular, they felt that it could reduce patients’ hospitalization and increase patients’ self-care, but they also identified a wide range of barriers for implementing telemonitoring at the level of organization, health care professionals, and patients.

When hospitals using telemonitoring for the purpose of clinical trials were excluded, we found that only one hospital in Japan used telemonitoring, and in Sweden it was not used at all. This was surprising since Japan and Sweden both have highly developed information technology and a high number of elderly HF patients [13,14]. In addition, Sweden has a long tradition of HF disease management programs. For these reasons, we assumed that many hospitals in the two countries had introduced a telemonitoring system in daily practice. However, our findings were completely different, especially when comparing them with a recently published study from the Netherlands, where 36% of hospitals were using telemonitoring [11] (and this was still considered a low figure). No specific number on the uptake of telemonitoring in other countries is known, although in several European countries (eg, Poland, Germany, and the United Kingdom) research studies on the effect of telemonitoring have been performed [3,5,19].

Despite the low use of telemonitoring, health care professionals could imagine a place for this technology among HF patients. They expected telemonitoring to reduce hospitalization rates, increase patient self-care, and high quality of care. These potential purposes for telemonitoring were also described by de Vries et al as expectations of telemonitoring in the Netherlands [11]. In that study, these expectations were not met after introducing telemonitoring, triggering us to focus on realistic and unrealistic expectations before the actual implementation. If expectations are high and are not met, this might lead to frustration and a waste of resources. Similar to the Dutch study [11], most of the health care staff in our study reported that an important aim of using telemonitoring would be to monitor the physical condition of the patient and to monitor and adjust treatment after remote drug titration. In our survey, the latter aim was less often viewed as applicable by the Japanese nurses, compared to the Swedish HF nurses. This can be explained by differences in the health care system between Sweden and Japan. Although citizens of both countries have basic health insurance and patients can have easy access to HF clinics and hospitals, there are some differences in the organization of care, for example, Swedish nurses are allowed to be active in drug titration, whereas nurses in Japan are not. Therefore, it is important to consider differences and similarities in health care systems and cultures in order to develop HF telemonitoring models.

Consistent with previous studies [11,20,21], the organization was identified as a significant barrier to the implementation of HF telemonitoring. Lack of structure, such as clear descriptions of responsibilities and protocols, as well as a lack of resources, such as manpower and materials, were seen as barriers standing in the way of using telemonitoring in current practice. In general, organizational issues are a key in the implementation of comprehensive health care systems [22]. A lack of a clear protocol describing actions to be taken based on the data received from the device can make telemonitoring time-consuming or make the health care professional feel insecure [20]. With regard to organization, previous studies have described that health care professionals may expect telemonitoring to be one of the solutions for medical staff shortages [20]. However, some studies have reported that telemonitoring is perceived as more laborious and as increasing one’s workload [23,24]. These findings suggest that the structure and resources in the organization go hand in hand, and therefore, implementation of telemonitoring should be considered as an organizational development and not merely a technical project.

Another barrier for implementation was the health care professionals’ reserved attitude. Not all physicians and nurses could see the advantage of using telemonitoring in their current practice, whereas others were skeptical about telemonitoring or reported that they did not know enough about it. These barriers are partly in line with barriers described in other studies [20,25]. Lack of relative advantage represents the degree to which a technology is perceived to be better than the existing alternatives [26]. It is one of the most important factors for adopting new technology [27]. In the case of telemonitoring in HF, it should be recognized that health care professionals might be confronted with mixed messages from research and industrial companies. A systematic review concluded that there was clear evidence of the clinical benefits in HF telemonitoring [2-4], while recent findings have not been consistent [5-8]. More evidence is still required on clinical benefits as well as the cost-effectiveness from a societal perspective. The finding that telemonitoring was not chosen as the best way to follow up on stable HF patients might be related to the perception of lack of advantage. Some industrial companies provide education and training, but a systematic educational support system regarding the concept, aims, and patient selection is necessary. Technology alone is not enough to create joined-up care pathways, and there is a need for continuous education of health care professionals at all stages of care.

Health care providers also had great doubts about the applicability of telemonitoring to certain patient groups or patients with certain characteristics or shortcomings. These included doubts about giving telemonitoring equipment to older patients, patients with physical and psychosocial impairments, or patients with no Internet/computer and who lacked computer skills. It is important to note that these are barriers as perceived by health care professionals, who had no experience of and/or lacked knowledge about HF telemonitoring. Thus, many of these barriers might be overcome by increased knowledge about telemonitoring and HF trajectory, but also clear instructions on how to use the devices and by improved design of equipment. Health care providers also expressed the concern that patients might get worried or anxious when using telemonitoring. However, these fears may disappear once they are using telemonitoring, as was shown in a study where such fear was reduced after patients began using telemonitoring devices. Patients had increased assurance and a sense of security [28,29]. Telemonitoring has been shown to enhance patients’ self-management, but our findings indicate that further work is required to develop an approach on how to increase patients’ acceptance and motivation to use HF telemonitoring.

### Limitations

There were several limitations to consider in this study. First, questions about barriers for implementing telemonitoring were asked, using open-ended question, which might limit our findings compared to using interviews. However, answers from nearly 500 health care providers were obtained. As for content analysis, all subcategories, categories, and themes were reviewed again to enhance conformability and minimize personal bias, which ensures trustworthiness of the findings. Second, as only a few hospitals had introduced telemonitoring for HF patients, it was not possible to describe health care professionals’ experiences of working with telemonitoring. Third, the instrument used was developed on the basis of a previous, validated Dutch study. However, some items might need to be modified considering the low percentage of health care institutions that had introduced HF telemonitoring in Japan and Sweden.

### Conclusions

This study demonstrated that only a few Japanese hospitals and no Swedish hospitals/clinics have introduced non-invasive telemonitoring for HF patients. Only a quarter of health care professionals were familiar with HF telemonitoring. However, cardiologists and nurses in Japan and Sweden expected HF telemonitoring to reduce patients’ hospitalization and increase patient self-care. There was, however, a wide range of barriers to implementing HF telemonitoring at the organizational, health care professional, and patient levels.

## Appendix

Multimedia Appendix 1 A questionnaire in the study.

## Abbreviations

HF: heart failure

## References

[1] Institute of Medicine (US) Committee on Evaluating Clinical Applications of Telemedicine (1996). Telemedicine: a guide to assessing telecommunications in health care.

[2] Klersy C, De SA, Gabutti G, Regoli F, Auricchio A (2009). A meta-analysis of remote monitoring of heart failure patients. J Am Coll Cardiol.

[3] Dar O, Riley J, Chapman C, Dubrey SW, Morris S, Rosen SD, Roughton M, Cowie MR (2009). A randomized trial of home telemonitoring in a typical elderly heart failure population in North West London: results of the Home-HF study. Eur J Heart Fail.

[4] Inglis SC, Clark RA, McAlister FA, Ball J, Lewinter C, Cullington D, Stewart S, Cleland JG (2010). Structured telephone support or telemonitoring programmes for patients with chronic heart failure. Cochrane Database Syst Rev.

[5] Koehler F, Winkler S, Schieber M, Sechtem U, Stangl K, Böhm M, Boll H, Baumann G, Honold M, Koehler K, Gelbrich G, Kirwan B, Anker M, Telemedical Interventional Monitoring in Heart Failure Investigators (2011). Impact of remote telemedical management on mortality and hospitalizations in ambulatory patients with chronic heart failure: the telemedical interventional monitoring in heart failure study. Circulation.

[6] Chaudhry SI, Mattera JA, Curtis JP, Spertus JA, Herrin J, Lin Z, Phillips CO, Hodshon BV, Cooper LS, Krumholz HM (2010). Telemonitoring in patients with heart failure. N Engl J Med.

[7] Dendale P, De KG, Troisfontaines P, Weytjens C, Mullens W, Elegeert I, Ector B, Houbrechts M, Willekens K, Hansen D (2012). Effect of a telemonitoring-facilitated collaboration between general practitioner and heart failure clinic on mortality and rehospitalization rates in severe heart failure: the TEMA-HF 1 (TElemonitoring in the MAnagement of Heart Failure) study. Eur J Heart Fail.

[8] Boyne JJ, Vrijhoef HJM, Crijns HJ, De Weerd G, Kragten J, Gorgels APM (2012). Tailored telemonitoring in patients with heart failure: results of a multicentre randomized controlled trial. Eur J Heart Fail.

[9] McMurray JJV, Adamopoulos S, Anker SD, Auricchio A, Böhm M, Dickstein K, Falk V, Filippatos G, Fonseca C, Gomez-Sanchez MA, Jaarsma T, Køber L, Lip GYH, Maggioni AP, Parkhomenko A, Pieske BM, Popescu BA, Rønnevik PK, Rutten FH, Schwitter J, Seferovic P, Stepinska J, Trindade PT, Voors AA, Zannad F, Zeiher A, Bax JJ, Baumgartner H, Ceconi C, Dean V, Deaton C, Fagard R, Funck-Brentano C, Hasdai D, Hoes A, Kirchhof P, Knuuti J, Kolh P, McDonagh T, Moulin C, Popescu BA, Reiner Z, Sechtem U, Sirnes PA, Tendera M, Torbicki A, Vahanian A, Windecker S, McDonagh T, Sechtem U, Bonet LA, Avraamides P, Ben Lamin HA, Brignole M, Coca A, Cowburn P, Dargie H, Elliott P, Flachskampf FA, Guida GF, Hardman S, Iung B, Merkely B, Mueller C, Nanas JN, Nielsen OW, Orn M, Parissis JT, Ponikowski P, Task Force for the Diagnosis and Treatment of Acute and Chronic Heart Failure 2012 of the European Society of Cardiology, ESC Committee for Practice Guidelines (2012). ESC guidelines for the diagnosis and treatment of acute and chronic heart failure 2012: The Task Force for the Diagnosis and Treatment of Acute and Chronic Heart Failure 2012 of the European Society of Cardiology. Developed in collaboration with the Heart Failure Association (HFA) of the ESC. Eur J Heart Fail.

[10] Dierckx R, Pellicori P, Cleland JGF, Clark AL (2015). Telemonitoring in heart failure: Big Brother watching over you. Heart Fail Rev.

[11] de Vries AE, van der Wal MHL, Nieuwenhuis MMW, de Jong RM, van Dijk RB, Jaarsma T, Hillege HL (2013). Health professionals' expectations versus experiences of internet-based telemonitoring: survey among heart failure clinics. J Med Internet Res.

[12] Fairbrother P, Ure J, Hanley J, McCloughan L, Denvir M, Sheikh A, McKinstry B, Telescot PT (2014). Telemonitoring for chronic heart failure: the views of patients and healthcare professionals - a qualitative study. J Clin Nurs.

[13] Zarrinkoub R, Wettermark B, Wändell Per, Mejhert M, Szulkin R, Ljunggren G, Kahan T (2013). The epidemiology of heart failure, based on data for 2.1 million inhabitants in Sweden. Eur J Heart Fail.

[14] Okura Y, Ramadan MM, Ohno Y, Mitsuma W, Tanaka K, Ito M, Suzuki K, Tanabe N, Kodama M, Aizawa Y (2008). Impending epidemic: future projection of heart failure in Japan to the year 2055. Circ J.

[15] Hsieh H, Shannon SE (2005). Three approaches to qualitative content analysis. Qual Health Res.

[16] Atkin P, Barrett D (2012). Benefits of telemonitoring in the care of patients with heart failure. Nurs Stand.

[17] Cyber Cross Japan Co.

[18] Kotooka N, Asaka M, Sato Y, Kinugasa Y, Nochioka K, Mizuno A, Nagatomo D, Mine D, Yamada Y, Eguchi K, Hanaoka H, Inomata T, Fukumoto Y, Yamamoto K, Tsutsui H, Masuyama T, Kitakaze M, Inoue T, Shimokawa H, Momomura S, Seino Y, Node K, HOMES- HI (2013). Home telemonitoring study for Japanese patients with heart failure (HOMES-HF): protocol for a multicentre randomised controlled trial. BMJ Open.

[19] Mortara A, Pinna GD, Johnson P, Maestri R, Capomolla S, La Rovere MT, Ponikowski P, Tavazzi L, Sleight P (2009). Home telemonitoring in heart failure patients: the HHH study (Home or Hospital in Heart Failure). Eur J Heart Fail.

[20] Boyne JJ, Vrijhoef HJM (2013). Implementing telemonitoring in heart failure care: barriers from the perspectives of patients, healthcare professionals and healthcare organizations. Curr Heart Fail Rep.

[21] Odeh B, Kayyali R, Nabhani-Gebara S, Philip N (2014). Implementing a telehealth service: nurses' perceptions and experiences. Br J Nurs.

[22] Berg M (2001). Implementing information systems in health care organizations: myths and challenges. Int J Med Inform.

[23] Lemay G, Azad N, Struthers C (2013). Utilization of home telemonitoring in patients 75 years of age and over with complex heart failure. J Telemed Telecare.

[24] Taylor J, Coates E, Brewster L, Mountain G, Wessels B, Hawley MS (2015). Examining the use of telehealth in community nursing: identifying the factors affecting frontline staff acceptance and telehealth adoption. J Adv Nurs.

[25] Crundall-Goode A, Goode KM (2014). Using telehealth for heart failure: Barriers, pitfalls and nursing service models. British Journal of Cardiac Nursing.

[26] Rogers Everett M (2003). Diffusion of Innovations. 5th ed.

[27] Greenhalgh T, Robert G, Macfarlane F, Bate P, Kyriakidou O (2004). Diffusion of innovations in service organizations: systematic review and recommendations. Milbank Q.

[28] Seto E, Leonard KJ, Cafazzo JA, Barnsley J, Masino C, Ross HJ (2012). Perceptions and experiences of heart failure patients and clinicians on the use of mobile phone-based telemonitoring. J Med Internet Res.

[29] Lyngå P, Fridlund B, Langius-Eklöf A, Bohm K (2013). Perceptions of transmission of body weight and telemonitoring in patients with heart failure?. Int J Qual Stud Health Well-being.

