# Supplementary material for: Heart Failure Telemonitoring in Japan and Sweden: A Cross-Sectional Survey
Source: J Med Internet Res. 2015 Nov 13;17(11):e258. doi: 10.2196/jmir.4825 (PMC4704966; doi:10.2196/jmir.4825)
Supplement: Multimedia Appendix 1 [file jmir_v17i11e258_app1.pdf]

For

Experiences with and opinions on using telemonitoring

This questionnaire concerns your experiences and opinion of telemonitoring when treating patients with heart failure.

Filling in the questionnaire will take about 15 minutes.

Definition

Telemonitoring

In this Questionnaire telemonitoring refers to remote, Internet-based monitoring, and monitoring of weight, blood pressure, heart rate, and signs and symptoms that disclose the actual condition of heart failure patients. The devices are used by the patients in their own home environment and the generated data are transferred by the Internet.

The use of telemonitoring by means of *telephone, telephone support, telephone follow-up, or by means of implantable device*s is ***not included*** in this study required an active user interaction.

<General Questions>

Please fill in or mark the answer that best applies to you for the general questions listed below.

1 *What is your gender?*

Male
Female

2 *How old are you?*

….. Years

3 *How many years have you been in your current position?*

….. years

4 *How many hours a week do you work with heart failure patients?*

….. hours
I don't know

5. *What is type of your hospital/institution?*

University hospital
General hospital
Other…, namely ....

6 *How many years of computer experience do you have (both work and private)?*

….. years

7. Do you have experience with programmes such as Word, PowerPoint, Excel or ITunes?

No
Yes

8. Do you have experience with e-mail?

No
Yes

9. Do you have experience with the internet?

No
Yes

10. Do you use e-mail on your mobile phone?

No
Yes

11 Which according to you would be good ways of performing follow-up of stable HF patients? The participants could chose more than one answer.

By telemonitoring using remote, internet-based monitoring
By implanted device
By phone
By video contact (for ex. Skype)
By e-mail on mobile phone
Outpatient clinic
Home visits by nurse
Home visit by other person
Other, namely ………..

<Telemonitoring>

12. Are you familiar with heart failure telemonitoring?

No

Yes

13. Does your heart failure clinic/cardiology clinic use telemonitoring?

No

Yes

14. Please indicate your opinion on a scale of 0 to 10 how important the following reasons were/might be to introduce telemonitoring to your centre in the future (0 = completely unimportant, 10 = very important)

**Importance of introducing telemonitoring**	**Mark between 0 and 10**
1. Offering higher-quality care	
2. Reducing costs	
3. Implementing the vision of the hospital	
4. Ability to treat more patients	
5. Reducing the workload on the heart failure out-patient clinic	
6. Reduce admissions/readmissions	
7. Better able to adherence with the heart failure guidelines	
8. Increasing the self-care of heart failure patients	
9. Our centre is innovative	
10. We want to keep up with developments in the field of telemonitoring	
11. It's mainly our health insurance company that thinks it's important	

15. What is/do you think might in future be a purpose for telemonitoring of your heart failure patients? (more than one answer possible)

Monitoring physical condition / signalling decline
Monitoring the effect of the treatment and adjusting it remotely
Remote drug titration
Patient education
Other, namely…………………………………………………….

16. Which telemonitoring system does your heart failure clinic use? (only for users of telemonitoring)

Name of the system/company …………………………………………………..

17. What are the important barriers to use telemonitoring in your institutions?
(only for non-users of telemonitoring)

………………………………………………………………………………………………
………………………………

This is the end of the questionnaire
Thank you.

**, The questions from #14 to # 16 were split for user and non-users of telemonitoring in the actual questionnaire.*
